# The Treatment of Uterine Displacements

**Published:** 1884-12

**Authors:** 


### BOOK NOTICES.

The Treatment of Uterine Displacements. By W. Eggert, M. D. Second edition. Pp. 136. Chicago: Duncan Brothers, 1884.

The treatment of uterine displacements by internal medication! How our incredulous friends—the specialists—must smile at the idea of such attempts. And when this internal medication is proclaimed to consist of a few doses of some inert remedy, say Sepia, in an high potency, then, indeed, must the *smile be very audible!*

Yet, however impossible for good such treatment may seem, it is nevertheless a *reliable* and successful one. Time and again has it been so proven. But to cure a displacement of the uterus, one must prescribe for the *patient*, and not simply for a displaced organ.

In confirmation of this method of treatment, we have this testimony of Dr. Skinner—a pupil of the great Simpson: "Since I have adopted Homœopathy I have never had occasion to introduce a pessary. With such medicines as Belladonna, Calcarea, Calcarea-phos., Conium, Kali-carb., Lachesis, Lilium, Lycopodium, Nux-vomica, Platina, Rhus-tox., Secale, Sepia, Sulphur, Thuja, Zinc, and a few others, we may safely consider ourselves equal to the cure of almost every conceivable case of pain or inconvenience from uterine displacement, without mechanical or local treatment of any kind."

In this useful little volume, Dr. Eggert gives the therapeutics of our best proven remedies, and lastly a brief and useful repertory, relating to these troubles.

